# Histone Demethylase Activity of Utx Is Essential for Viability and Regulation of *HOX* Gene Expression in *Drosophila*

**DOI:** 10.1534/genetics.117.300421

**Published:** 2017-12-14

**Authors:** Ömer Copur, Jürg Müller

**Affiliations:** Laboratory of Chromatin Biology, Max Planck Institute of Biochemistry, 82152 Martinsried, Germany

**Keywords:** Polycomb, trithorax, Utx, H3K27me3 demethylation, *Drosophila*

## Abstract

The trimethylation of histone H3 at lysine 27 (H3K27me3) by Polycomb Repressive Complex 2 (PRC2) is essential for the repression of Polycomb target genes. However, the role of enzymatic demethylation of H3K27me3 by the KDM6-family demethylases Utx, Uty, and JmjD3 is less clear. Studies in both mice and worms led to the proposal that KDM6 proteins, but not their H3K27me3 demethylase activity, is critical for normal development. Here, we investigated the requirement of the demethylase activity of the single KDM6 family member Utx in *Drosophila*. We generated *Drosophila* expressing a full-length but catalytically inactive Utx protein and found that these mutants show the same phenotypes as animals lacking the Utx protein. Specifically, animals lacking maternally deposited active Utx demethylase in the early embryo show stochastic loss of HOX gene expression that appears to be propagated in a clonal fashion. This suggests that Utx demethylase activity is critical for the removal of ectopic H3K27 trimethylation from active HOX genes during the onset of zygotic gene transcription, and thereby prevents the inappropriate installment of long-term repression by Polycomb. Conversely, maternally deposited catalytically active Utx protein suffices to permit animals that lack zygotic expression of enzymatically active Utx to develop into morphologically normal adults, which eclose from the pupal case but die shortly thereafter. Utx demethylase activity is therefore also essential to sustain viability in adult flies. Together, these analyses identify the earliest embryonic stages and the adult stage as two phases during the *Drosophila* life cycle that critically require H3K27me3 demethylase activity.

CHROMATIN modifications have emerged as central mechanisms by which Polycomb Group (PcG) and trithorax Group (trxG) protein complexes regulate the expression of genes that control development in animals and plants. A key step for Polycomb repression is the trimethylation of histone H3 at lysine 27 (H3K27me3), which Polycomb Repressive Complex 2 (PRC2) deposits across extended regions of chromatin at repressed genes. H3K27me3 is thought to mark nucleosomes for interaction with PRC1, another PcG protein complex. The central role of this methylation is illustrated by the finding that a histone H3K27R point mutation in *Drosophila* reproduces the phenotype of mutants lacking PRC2 or PRC1 function (Pengelly *et al.* 2013; McKay *et al.* 2015). H3K27me3 appears to be a stable modification and H3K27me3-containing nucleosomes have been shown to be transmitted to daughter-strand DNA during DNA replication (Alabert *et al.* 2015; Coleman and Struhl 2017; Laprell *et al.* 2017). However, such transmitted H3K27me3-modified nucleosomes provide only a short-term memory for Polycomb repression; long-term maintenance of this repression requires that PRC2 methylates K27 on the newly incorporated H3 histones after every replication cycle (Coleman and Struhl 2017; Laprell *et al.* 2017). Cell division-coupled dilution of H3K27me3 in the absence of methylation by PRC2 would therefore provide a possible mechanism for converting a previously Polycomb-repressed gene into an active gene.

About a decade ago, members of the lysine-specific demethylase 6 (KDM6) family of histone demethylases were identified as enzymes that remove the H3K27me3 mark from nucleosomes (Agger *et al.* 2007; De Santa *et al.* 2007; Hong *et al.* 2007; Lan *et al.* 2007; Lee *et al.* 2007; Walport *et al.* 2014). This discovery suggested that active demethylation of H3K27me3 might provide an alternative mechanism for activating Polycomb-repressed genes. The number of KDM6 paralogs varies between species. The best-characterized KDM6 family member in animals is Utx (also known as Ubiquitously transcribed tetratricopeptide repeat protein, X chromosome), a subunit of the MLL3/4 complex in mammals and the orthologous Trithorax-related complex in flies (Cho *et al.* 2007; Issaeva *et al.* 2007; Lee *et al.* 2007; Mohan *et al.* 2011). Genetic studies showed that Utx and other KDM6 family proteins have important functions during the development of mice, flies, and worms. However, the role of their H3K27me3 demethylase activities for these different functions is not well understood. First, in mice, females homozygous for a deletion allele of the X-linked Utx gene die as early embryos, but hemizygous males develop into viable and fertile adults. The lack of a phenotype in males has been ascribed to compensation by the Y-linked KDM6 protein Uty (also known as Ubiquitously Transcribed Tetratricopeptide Repeat-Containing protein, Y-Linked), which has only poor catalytic activity *in vitro* and appears largely inactive *in vivo* (Shpargel *et al.* 2012; Walport *et al.* 2014). Consequently, the Utx mutant phenotype in female mice was proposed to be due to the lack of Utx protein and not a lack of its demethylase activity (Shpargel *et al.* 2012). Second, male mice lacking both Utx and the KDM6 paralogue Jmj domain-containing protein 3 (JmjD3) do not show more severe phenotypes than JmjD3 single mutants, suggesting that the two proteins do not act in a redundant fashion (Shpargel *et al.* 2014). Finally, Utx catalytic activity was reported to be dispensable for mesoderm differentiation in an embryonic stem cell model *in vitro* (Wang *et al.* 2012). Collectively, these studies have therefore questioned the functional relevance of H3K27me3 demethylation by KDM6 proteins in mice. Similarly, in *C. elegans*, UTX-1 is essential for viability, but this phenotype was fully rescued by a transgene expressing a catalytically inactive UTX-1 protein, even in animals that also lacked the three JMJD3 paralogs (Vandamme *et al.* 2012). This led to the conclusion that H3K27me3 demethylation by KDM6 proteins is also dispensable in worms.

Unlike in *Caenorhabditis elegans* and mice, *Drosophila* only contains a single KDM6 ortholog, called *dUtx* (Smith *et al.* 2008; Herz *et al.* 2010). *Drosophila* *Utx* null mutants that contain maternally deposited Utx protein during the early stages of embryogenesis develop into adults that show normal morphology but die shortly after eclosing (Copur and Müller 2013). However, animals lacking both maternally deposited and zygotically expressed Utx protein die during larval development, and they fail to maintain expression of multiple HOX genes in different tissues (Copur and Müller 2013). Such *Utx* null mutant animals therefore show classic trxG phenotypes. In this study, we used a genetic rescue strategy to investigate whether these phenotypes are due to the lack of Utx H3K27me3 demethylase activity. Our analyses show that Utx catalytic activity is essential for the viability of *Drosophila* and plays a prominent role in the regulation of HOX gene expression.

## Materials and Methods

A genomic BAC clone (CH321-26E08) that contains the entire *Utx* gene was obtained from the BACPAC Resources Center. The wild-type *gUtx^wt^* rescue construct contained sequences from the CH321-26E08 BAC clone, which we amplified with the 5′-GACGGTACCCAGGGCTACACCAATATCAACCAATTG-3′ and 5′-GATCGGATTCCCGAGCAAACACATCTAAGGCCAAAGGAG-3′ primers (genomic coordinates R1.17 chr2L: 10272223–10279218) and subcloned into a TOPO cloning vector (ThermoFisher). To generate g*Utx^cd^*, the CAC and GAG codons for His883 and Glu885, respectively, were mutated to GCC and GCG, respectively, to generate the His883Ala, Glu885Ala mutant. g*Utx^wt^* and g*Utx^cd^* DNA inserts in the TOPO vector were then cloned into an attB vector and inserted into the 86Fb attP landing site by using the φC31-based integration method.

### *Drosophila* strains

The following *Drosophila* strains were used and/or generated for this study:

Oregon-R.
w; Utx ^Δ^ FRT40A/ Cyo,twi::GFP.
w; Df(2L)BSC143/ Cyo,twi::GFP.
w; Utx^Δ^ FRT40A/ Cyo,twi::GFP; gUtx^wt^.
w; Df(2L)BSC143/ Cyo,twi::GFP; gUtx^wt^.
w; Utx^Δ^ FRT40A/ Cyo,twi::GFP; gUtx^cd^.
w; Df(2L)BSC143/ Cyo,twi::GFP; gUtx^cd^.
yw, hsflp; ovo^D1^ FRT40A/ CyO, hs::hid.
yw, hsflp; ovo^D1^ FRT40A/ CyO, hs::hid; gUtx^wt^.
yw, hsflp; Utx^Δ^ FRT40A/ CyO, hs::hid; gUtx^wt^.
yw, hsflp; ovo^D1^ FRT40A/ CyO, hs::hid; gUtx^cd^.
yw, hsflp; Utx^Δ^ FRT40A/ CyO, hs::hid; gUtx^cd^.

### Western blot analysis and immunostaining procedures

Western blot analysis of larval tissues and immunostaining of imaginal discs was performed as described (Copur and Müller 2013). The following antibodies were used: anti-rabbit Utx (1:1000) (Tie *et al.* 2012), anti-rabbit Caf-1 (1:20000) (Gambetta *et al.* 2009), and anti-mouse Ubx (1:30) (Developmental Studies Hybridoma Bank).

### Data availability

Transgene DNA and *Drosophila* strains generated in this study are available upon request.

## Results and Discussion

**Figure 1:**
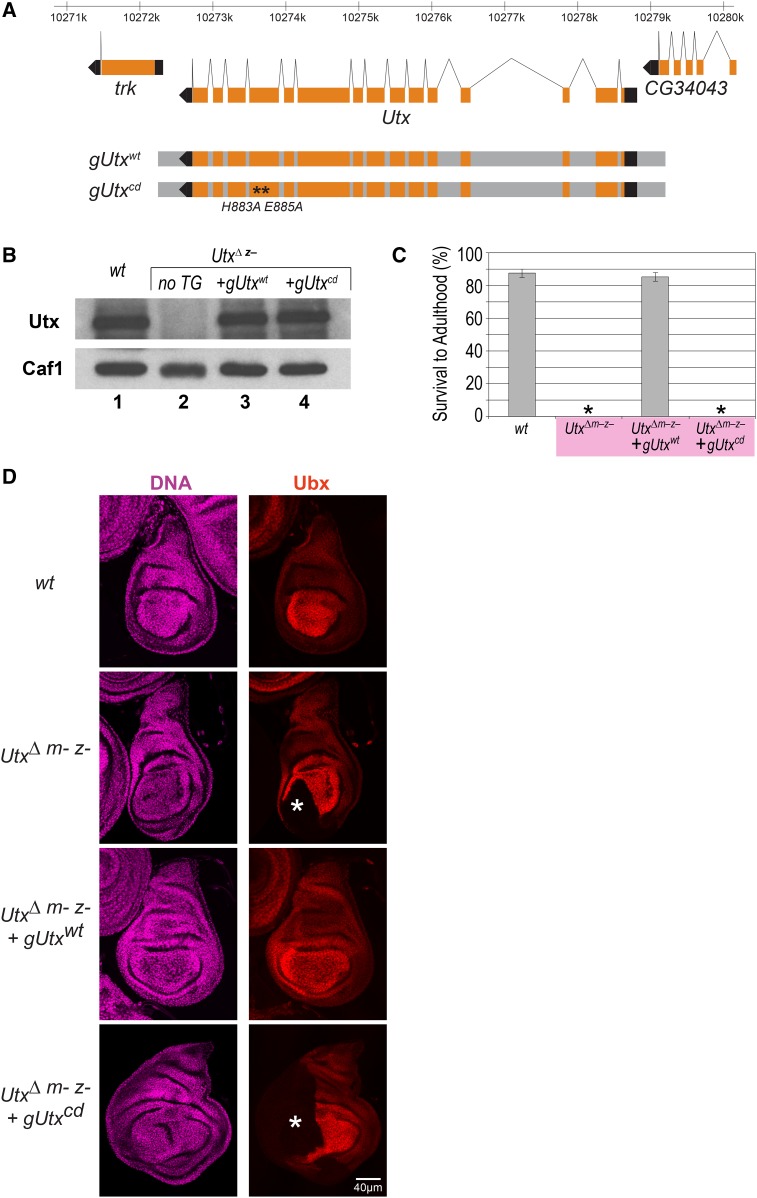
Utx demethylase activity is critical for viability and HOX gene regulation. (A) Top: view of the genomic region harboring *Utx* and flanking genes with coding (orange boxes) and noncoding exons (black boxes), and introns indicated above. Below: genomic fragments (gray bars) used for *gUtx^wt^* and g*Utx^cd^* transgenes; asterisks indicate locations of the codons for His883 and Glu885 that were mutated to codons for Ala in g*Utx^cd^*. (B) Western blot analysis of total extracts from imaginal disc and central nervous system tissues from third-instar larvae of the indicated genotypes probed with antibodies against Utx protein and, as loading control, Caf1 protein. Note that in *Utx*^Δ^ homozygotes (*Utx*^Δ^ *^z^*^–^), the transgene-encoded Utx^wt^ and Utx^cd^ proteins (lanes 3 and 4) are present at levels comparable to the levels of Utx protein in wild-type (*wt*) larvae (lane 1). (C) Survival of *wt* animals and of animals of the indicated genotypes to adulthood. *Utx*^Δ^ *^m– z^*^–^ animals obtained using the *ovo^D^* technique all arrest development before reaching the pupal stage (0 animals surviving into adults, asterisk). *Utx*^Δ^ homozygous animals carrying the *gUtx^wt^* transgene (*Utx*^Δ^ *^m– z–^ + gUtx^wt^*) can be maintained as a healthy strain with survival rates comparable to *wt*. *Utx*^Δ^ *^m– z^*^–^ animals carrying the *gUtx^cd^* transgene (*Utx*^Δ^ *^m– z–^ + gUtx^cd^*), obtained using the *ovo^D^* technique, all arrest development before reaching the pupal stage (0 animals surviving into adults, asterisk). For each genotype, the value of survival to adulthood was calculated by collecting three independent batches with of 100 first instar larvae, transferring the three batches of larvae into independent vials and determining the number of eclosed adults in each vial. Survival to adulthood values (%) represent mean values from the three batches and error bars indicate SD. (D) Haltere imaginal discs from larvae that were *wt* or the indicated *Utx* genotypes stained with antibody against Ubx protein (Ubx) and Hoechst (DNA). In *wt* animals, Ubx is expressed in all cells of the haltere disc with the strongest levels detected in cells in the pouch. In *Utx*^Δ^ *^m– z^*^–^ animals, Ubx protein is undetectable in clone-like large contiguous patches of cells (asterisk marks the single large patch seen in this disc), whereas adjoining cells express apparently undiminished levels of Ubx protein. In *Utx*^Δ^ *^m– z–^ + gUtx^wt^* animals, Ubx expression is indistinguishable from *wt* animals. *Utx*^Δ^ *^m– z–^ + gUtx^cd^* animals show loss of Ubx protein expression in clone-like large patches of cells (asterisk marks the single large patch seen in this disc), similar to *Utx*^Δ^ *^m– z^*^–^ animals.

The active site of the Fe(II)- and α-ketoglutarate-dependent dioxygenase Utx is highly conserved between flies and humans. Structural and biochemical studies on human UTX established that the His1146 and Glu1148 residues form the Fe(II)-binding motif, and that mutation of these two residues to Ala abolishes H3K27me3 demethylase activity of UTX *in vitro* (Sengoku and Yokoyama 2011). Moreover, mutation of the corresponding His914 and Asp916 residues in *C. elegans* UTX-1 was shown to abolish its demethylase activity *in vivo* (Vandamme *et al.* 2012). We analyzed the phenotype of *Drosophila* expressing a catalytically inactive Utx protein with the Fe(II)-binding His883 and Glu885 residues mutated to Ala, as follows. We generated transgenes containing a genomic *Utx* fragment expressing either the wild-type Utx protein (*gUtx^wt^*), or the catalytically inactive Utx protein with the His883Ala/Glu885Ala mutations (g*Utx^cd^*, Figure 1A). The *gUtx^wt^* and *gUtx^cd^* transgenes were then introduced into the genetic background of animals carrying a deletion of the endogenous Utx locus (*Utx*^Δ^). As previously reported, *Utx*^Δ^ animals lack detectable levels of full-length Utx protein (Figure 1B, lanes 1 and 2; Copur and Müller 2013). Importantly, the transgene-encoded wild-type and Utx^cd^ mutant proteins were both expressed at wild-type levels in such *Utx*^Δ^ animals (Figure 1B, lanes 3 and 4).

We first analyzed the requirement for Utx catalytic activity for adult viability. *Utx*^Δ^ homozygous animals derived from heterozygous mothers (*Utx*^Δ^ *^z^*^–^) die as adults, shortly within 1–2 days after eclosing from the pupal case. This phenotype was fully rescued by the Utx^wt^ but not by the catalytically inactive Utx^cd^ protein. Specifically, *Utx*^Δ^ homozygous animals carrying the *gUtx^wt^* transgene (*Utx*^Δ^ *gUtx^wt^*) were viable, fertile, and could be maintained as a healthy strain. In contrast, *Utx*^Δ^ *gUtx^cd^* animals developed into morphologically normal-looking adults that died within 1–2 days after eclosing from the pupal case (*N* > 500), like *Utx*^Δ^ animals carrying no transgene (*N >* 500). This shows that Utx demethylase activity is essential for adult viability.

Previous studies demonstrated a critical role of Utx protein during the earliest stages of *Drosophila* embryogenesis (Copur and Müller 2013). Specifically, *Utx*^Δ^ mutant animals that are derived from females with *Utx*^Δ^ mutant germ cells and therefore lack not only zygotic expression of Utx but also maternally deposited Utx protein (*Utx*^Δ^ *^m– z^*^–^ animals) are unable to complete development and die as larvae (Copur and Müller 2013). Because the *Utx*^Δ^ *gUtx^cd^* animals described above still contained maternally deposited wild-type Utx protein, we next generated mutant embryos in which both the maternally deposited and the zygotically expressed Utx protein were catalytically inactive (*Utx*^Δ^ *^m– z–^ gUtx ^cd^*). As illustrated in Figure 1C, *Utx*^Δ^ *^m– z–^ gUtx ^cd^* animals failed to develop into adults and died during the larval stages, like *Utx*^Δ^ *^m– z^*^–^ animals (Figure 1C). As noted above, *Utx*^Δ^ *^m–z–^ gUtx^wt^* animals could be maintained as a healthy strain with survival rates comparable to wild-type (Figure 1C). We conclude that Utx demethylase activity is critically required for normal embryonic and larval development.

Next, we investigated whether Utx demethylase activity is required for the normal regulation of HOX gene expression. We monitored the expression of the HOX gene *Ultrabithorax* (*Ubx*) in *Utx*^Δ^ *^m– z^*^–^, *Utx*^Δ^ *^m– z–^ gUtx ^wt^*, and *Utx*^Δ^ *^m– z–^ gUtx ^cd^* mutant larvae. In wild-type larvae, Ubx protein is expressed in all cells of the haltere imaginal discs (Figure 1D). In *Utx*^Δ^ *^m– z^*^–^ mutant larvae, Ubx expression is lost in a patchy pattern from large areas of both haltere discs in each larva (*N* = 56 larvae; Figure 1D). The transgene-encoded Utx^wt^ protein restored the normal Ubx expression pattern in all *Utx*^Δ^ *^m– z–^ gUtx^wt^* animals (*N* = 45 larvae; Figure 1D). In contrast, the Utx^cd^ protein was unable to rescue the phenotype, and *Utx*^Δ^ *^m– z–^ gUtx ^cd^* animals showed the same patchy loss of Ubx expression as *Utx*^Δ^ *^m– z^*^–^ mutants (*N* = 52 larvae; Figure 1D). Utx demethylase activity is therefore important for normal HOX gene expression.

### Conclusions

An important genetic test to understand the mechanism of proteins that possess enzymatic activities is to validate that mutants expressing a catalytically inactive but otherwise intact form of the protein show the same phenotype as mutants lacking the protein. This is particularly critical for enzymatic subunits that are part of multi-protein assemblies where they often also play an architectural role, as is the case in many chromatin-modifying complexes. Here, we show that *Drosophila* expressing catalytically inactive but full-length Utx protein reproduce the phenotype of an *Utx* gene deletion mutant. Unlike in *C. elegans* (Vandamme *et al.* 2012), lack of Utx demethylase activity in *Drosophila* is deleterious to development and viability. Here, we focused on the role of Utx enzymatic activity for the regulation of the HOX gene *Ubx*, which we previously identified as a prominent target gene requiring *Utx* function (Copur and Müller 2013). Like in mutants lacking Utx protein, animals that lack Utx H3K27me3 demethylase activity during the earliest stages of embryogenesis develop into third-instar larvae that show loss of Ubx expression in imaginal disc tissues. The loss of Ubx expression in large contiguous regions of these tissues, juxtaposed to cells with apparently undiminished levels of expression (Figure 1D), implies that Ubx expression was stochastically lost in a fraction of cells early in development and that this loss was then clonally propagated. Recent studies have reported that H3K27me3 present at Polycomb target genes in the oocyte is maintained and propagated throughout the early cleavage cycles, and is required to prevent precocious transcription of these genes during zygotic gene activation at the blastoderm stage (Zenk *et al.* 2017). During subsequent development, in postblastoderm embryos, the H3K27me3 profiles are then resolved, as recently demonstrated at HOX genes; in cells where HOX genes are repressed, their chromatin becomes fully decorated with H3K27me3, thus enabling long-term Polycomb repression, and in cells where they are transcribed, their chromatin becomes devoid of H3K27me3 (Bowman *et al.* 2014). We propose that H3K27me3 demethylation by Utx is critical during the establishment of these H3K27me3-deficient chromatin states. We envisage that, in the absence of H3K27me3 demethylation by Utx, PRC2 also establishes high levels of H3K27me3 at target genes in some of the cells where these genes should be expressed. The stochastic loss of HOX gene expression in *Utx* mutants in those cells would therefore reflect aberrant installment of Polycomb repression, analogous to what has been observed in mutants that lack other trxG regulators (Klymenko and Müller 2004; Papp and Müller 2006).
